# Infrared Thermographic Imaging of Chest Wall Perfusion in Patients Undergoing Coronary Artery Bypass Grafting

**DOI:** 10.1007/s10439-022-02998-x

**Published:** 2022-06-30

**Authors:** Stefan Rasche, Christian Kleiner, Jens Müller, Antje Rost, Tamer Ghazy, Katrin Plötze, Ronald Tetzlaff, Klaus Matschke, Olimpiu Bota

**Affiliations:** 1 Surgical Intensive Care Unit, Faculty of Medicine Carl Gustav Carus, TU Dresden, Fetscherstraße 74, 01307 Dresden, Germany; 2 Institute of Acoustics and Speech Communication, TU Dresden, Dresden, Germany; 3 Faculty of Electrical and Computer Engineering, Institute of Circuits and Systems, TU Dresden, 01067 Dresden, Germany; 4 Department of Anesthesiology and Intensive Care Medicine, Faculty of Medicine Carl Gustav Carus, TU Dresden, Fetscherstraße 74, 01307 Dresden, Germany; 5 Department of Cardiac Surgery, Marburg University Hospital, Marburg, Germany; 6 Department of Cardiac Surgery, University Heart Center Dresden, TU Dresden, Fetscherstrasse 76, 01307 Dresden, Germany; 7 University Center for Orthopedics, Trauma and Plastic Surgery, Faculty of Medicine Carl Gustav Carus, TU Dresden, Fetscherstraße 74, 01307 Dresden, Germany

**Keywords:** Infrared thermography, Tissue perfusion, Coronary artery bypass, Internal mammary artery, Chest wall perfusion

## Abstract

Coronary artery disease represents a leading cause of death worldwide, to which the coronary artery bypass graft (CABG) is the main method of treatment in advanced multiple vessel disease. The use of the internal mammary artery (IMA) as a graft insures an improved long-term survival, but impairment of chest wall perfusion often leads to surgical site infection and increased morbidity and mortality. Infrared thermography (IRT) has established itself in the past decades as a non-invasive diagnostic technique. The applications vary from veterinary to human medicine and from head to toe. In this study we used IRT in 42 patients receiving CABG to determine the changes in skin surface temperature preoperatively, two hours, 24 h and 6 days after surgery. The results showed a significant and independent drop of surface temperature 2 h after surgery on the whole surface of the chest wall, as well as a further reduction on the left side after harvesting the IMA. The temperature returned to normal after 24 h and remained so after 6 days. The study has shown that IRT is sufficiently sensitive to demonstrate the known, subtle reduction in chest wall perfusion associated with IMA harvesting.

## Introduction

Coronary artery disease (CHD) represents a leading cause of death worldwide, to which the coronary artery bypass graft (CABG) is the main method of treatment in advanced multiple vessel disease. The procedure implies the completion of a median sternotomy and bypassing the diseased coronary artery segments with autologous vessels. The use of the internal mammary artery (IMA) as a graft insures an improved long-term survival.^12^ Nevertheless, the combination of median sternotomy with the harvest of the sternal blood supply, the internal mammary arteries, leads to a decrease in the regional blood perfusion, which predisposes to postoperative wound healing disorders and deep sternal wound infections (DSWI).^1^ To minimize the occurence of these complications, the harvest of only the left internal mammary artery (LIMA) is currently favored in most heart surgery centers,^15^ while harvesting both internal mammary arteries (BIMA) is used only in chosen cases.^4^An alternative is the harvesting of a skeletonized IMA, leaving the collateral circulation around the artery intact and therefore preserving some of the sternal perfusion.^6^

Patient comorbidities like obesity, diabetes mellitus, heart failure or a history of smoking may favor the hypoperfusion of the anterior chest area postoperatively.^19^ Therefore, the perioperative diagnosis of perfusion impairment around the chest wall is of great importance, especially in patients at risk for developing wound complications. Nevertheless, standard measures to estimate chest wall perfusion have not been yet defined.

The measurement of thermal radiation has been proven so far to have useful applications in different domains like monitoring the heart rate and respiratory rates in experimental pigs,^2^ brain mapping,^7,10,16^ tumor detection,^22^ identifying the perforator vessels for flap surgery^26,9^ as well as for determining the skin perfusion postoperatively after flap harvest.^20^ Infrared thermography (IRT) implies a non-invasive, accessible and label-free technique. In patients undergoing CABG surgery, high resolution thermography of the chest wall may be valuable for identifying changes in perfusion, especially in patients at risk.

In our study we aimed at estimating changes in tissue perfusion by means of IRT after the harvest of the LIMA for bypass surgery. Patient-related risk factors, surgical technique, vital parameters and cutaneous blood volume pulse (CBVP) were investigated for additional effects on IRT.

## Materials and Methods

### Study Setting

Patients who underwent CABG were studied at our university center for cardiac surgery, after written informed consent was obtained. The study was conducted in accordance with the principles of the Declaration of Helsinki, fifth revision, and was approved by the Institutional Review Board of our institution (IRB00001473, EK168052013). This was a combined study to evaluate both high resolution thermographic imaging as well as contact-free photoplethysmographic imaging (PPGI)^11^ for monitoring purposes in major/cardiac surgery.

### Thermography Setup

The chest wall surface temperature T_s_ was measured by the high resolution IRT system VarioCAM^®^ (InfraTec GmbH, Dresden, Germany). VarioCAM^®^ uses an uncooled microbolometric plane array with a resolution of 480 × 640 pixels. Its thermal resolution is 30 mK at 30 °C in a spectral range from 7.5 to 14 μm. Its spatial resolution reaches 0.25 mm with an object distance of 300 mm. Images are recorded with 30 frames per second.

### Measurements and Image Processing

Measurements were performed the day before surgery (BL) as well as two hours (H2), 24 h (H24) and 6 days (D6) after surgery. Measurements were taken in supine position. Patients were in stable circulatory condition and weaned from mechanical ventilation during the measurements after surgery.

**Figure 1:**
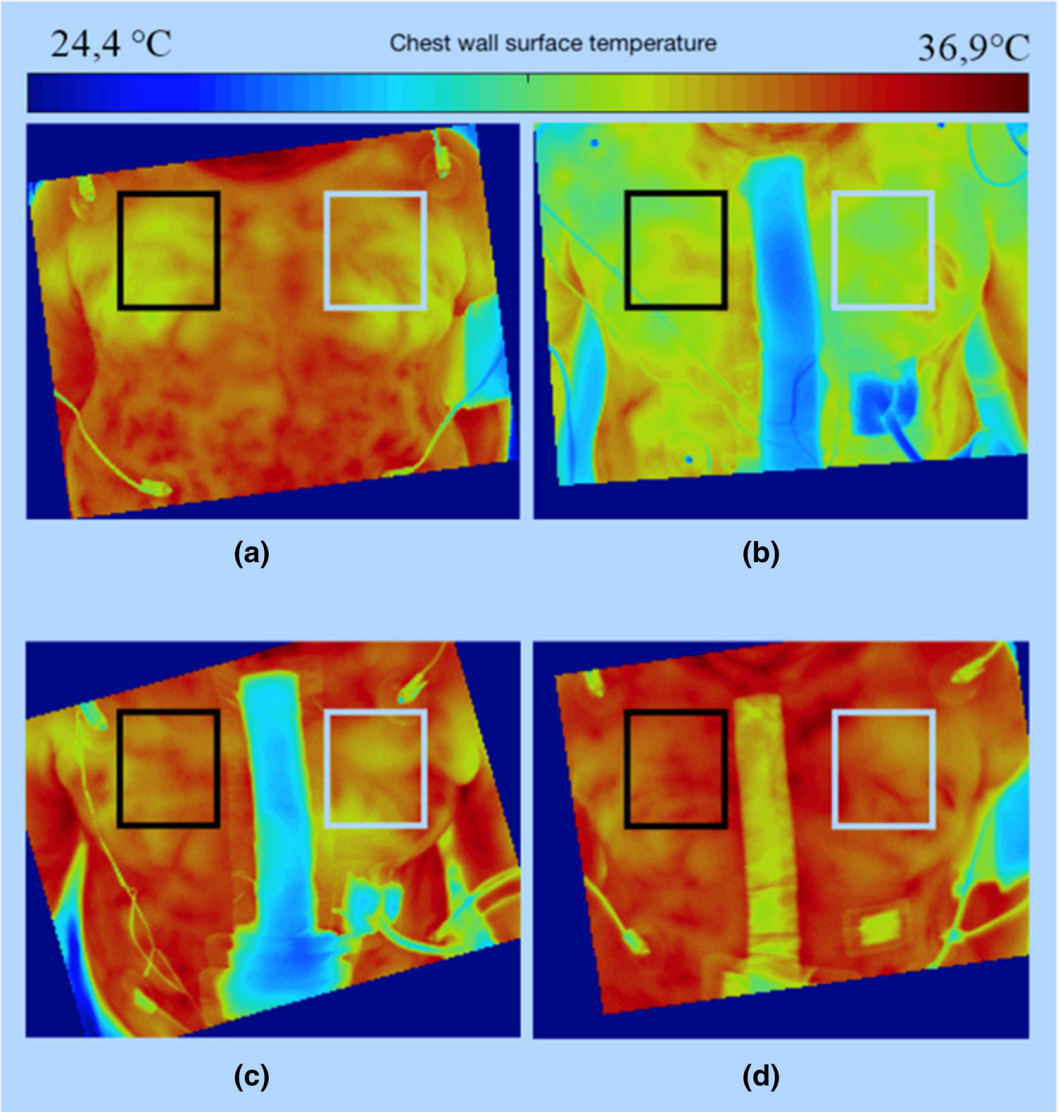
Thermal images of the chest wall with left (white) and right (black) ROI. (a) before surgery, (b) 2 h after surgery, (c) 24 h after surgery, (d) 6 days after surgery. All temperature values were averaged over 18,000 frames, corresponding to a duration of 60 s.

Before each measurement, an internal camera one-point non-uniformity correction (NUC) was performed for each microbolometer of the uncooled focal plane array to avoid a recalibration during data collection. A sequence of thermographic images of one minute duration was taken at each measurement. Data were processed offline after all measurements were completed. Consistent rectangular regions of interest (ROI) were defined for each side of the chest in the four videos, leaving out wound dressings and drains (Fig. 1). The temperatures within the ROI were averaged over time and space, leading to the two temperature values *T*_S,L_ and *T*_S,R_ for the left and right side of the anterior chest wall for each measurement. Blood pressure and heart rate, body (core) temperature and room temperature were taken for reference at each measurement. Surrogates of cutaneus perfusion were derived from the CBVP, which was estimated in non-contact photoplethysmograms of the accompanying study at the same time points.^11^

### Statistical Analysis

Effects of surgery and surgical techniques, vital parameters, and cofactors on T_s_ were investigated in random-intercept covariance models. Random effects were defined by patients. Fixed effects were defined by measurement time, chest wall side, vital signs, surgical factors and comorbidity. Maximal models containing covariates of any interest were constructed, pared down on the basis of stepwise deletion and compared by Likelihood ratio (LR) tests. Core temperature and room temperature variables were scaled to their grand mean. Heart rate was scaled to deviations of 10bpm from 80 bpm. Measurement time was defined as an ordinal variable in relation to surgery. Significance of fixed effects was t-tested after Satterthwaite´s approximation to degrees of freedom.^13^ The explanatory power of room and core temperature on *T*_s_ was taken as the reduction of the residual variance of *T*_s_ from the minimal adequate model through stepwise deletion of both predictors.

## Results

Fortytwo patients undergoing CABG surgery were investigated. Patient characteristics and graft preparation are given in Table 1.

**Table 1:** Demographic and baseline data.

Patients (*m*:*w*)	39:3
Age (years)	71.1 ± 7.5
Weight (kg)	87.3 ± 13.9
Height (cm)	174 ± 7.2
Arterial hypertension (*n*)	37
Graft preparation (ped:scel)	27:15
NYHA (n)	
1	2
2	18
3	22
LVEF	
Normal	26
Borderline	4
Impaired	8
Severely impaired	4
Diabetes (*n*)	18
Hyperlipidemia (*n*)	39
Angina pectoris (*n*)	18
CCS 1	5
CCS 2	6
CCS 3	4
CCS 4	3

*Ped* pedicled mammaria graft, *scel* skeletonized mammaria graft, *NYHA* New York heart Association functional class, *LVEF* preoperative left ventricular ejection fraction, *CCS* Canadian Cardiovascular Society grading of angina pectoris

### Impact of Room and Core Temperature

T_S_ varied between 28.8 and 35.8 °C and was on average 3.2 °C lower than core temperature (*p* < 0.001, *U* Test). *T*_S_ significantly increased with room temperature or core temperature in univariable and multivariable analyses. A varying impact of both parameters on *T*_s_ during the study was not evident after adjustment for cofactors. Independently from surgery and physiological parameters, room temperature explained 8.3% of the variance of *T*_s_, whereas body temperature explained 1.2% (*p* = 0.04) and both together explained 11.5% (*p* < 0.001).

### Surgery, Vital Parameters and Comorbidity

**Figure 2:**
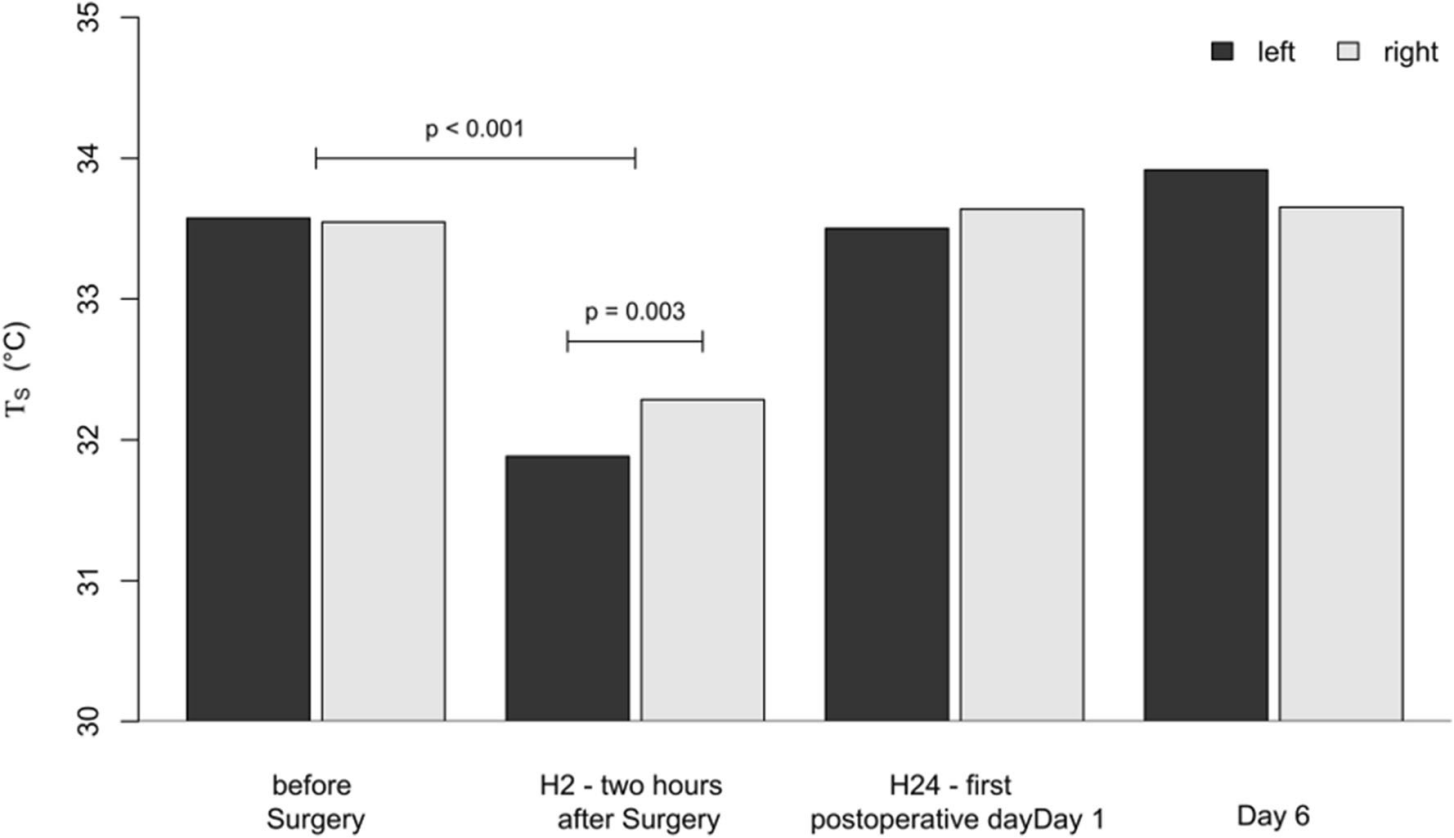
Surface Temperature *T*_S_ of the left and right side of the anterior chest wall during the study.

T_S_ significantly decreased immediately after surgery (H2) by 1.7 K (from − 2.0 to − 1.4 K, *p* < 0.001) at the left side and by 1.3 K from (− 1.6 K to − 0.9 K, *p* < 0.001) at the right side. It recovered the day after surgery (H24) and slightly exceeded the preoperative level on day 6. A difference in *T*_S_ of 404 mK (*p* = 0.015) between the left and right side was seen at H2, but neither before surgery nor at H24 or D6 (Fig. 2, Table 2). Core temperature also decreased after surgery (− 0.77 °C, *p* < 0.001). The measurement point *per se* remained significant after multivariable adjustment of related cofactors including core and room temperature.

**Table 2:** *T*_S_ of both sides of the chest during the study.

	${T}_{\text{s}}$_left_ (°C)	SD	${T}_{\text{s}}$_right_ (°C)	SD	${p}_{1}$	${p}_{2}$
Baseline	33.6	0.9	33.5	0.8	–	0.869
H2	31.9	1.1	32.3	1.0	< 0.001	0.015
H24	33.5	1.1	33.6	1.1	0.675	0.422
Day6	33.9	0.9	33.7	0.9	0.045	0.119

*SD* standard deviation. *p*_1_ changes over time, *p*2 left side *vs*. right side of the chest wall

Table 3 shows the multivariable estimation of concurrent effects of vital parameters, surgery and comobidity on *T*_S_. A significant impact of surgery on global *T*_S_ remains evident until the first postoperative day (H24). Heart rate affected *T*_S_ at BL, H24 and D6, but not at H2. At heart rates higher than 80 bpm, *T*_S_ increased by 0.3 °C (BL), 0.4 °C (H24) and 0.3 °C (D6) per 10 bpm increase of heart rate. A higher respiratory rate was associated with a slight decrease of *T*_S_. Sensitivity of *T*_S_ to blood pressure was only seen in the subgroup of patients with documented angina pectoris before surgery. Among those, patients with angina of Canadian Cardiovascular Society (CCS) class 4 had a significant delay in postoperative recovery of *T*_S_, compared to lower classes (Table 4). Preoperative systolic cardiac function (ejection fraction) and heart failure according to the New York Heart Associacion (NYHA) classification had no influence on *T*_S_. Likewise, arterial hypertension, diabetes mellitus or lipid disorders did not exert a significant effect on *T*_S_. Surgical techniques (pedicled *vs*. skeletonized grafting) and ancillary measures like the use of stapler and gelaspone or intracutaneous suture were not systematically associated with *T*_S_.

**Table 3:** Multivariable estimation of the effects of surgery vital parameters and comorbidity on T_S_.

	${\text{Estimate}}$	CI low	CI high	$p$
Effect of surgery				
H2	− 2.2	− 2.9	− 1.5	<0.001
H24	− 0.7	− 1.3	− 0.2	0.015
D6	0.1	− 0.3	0.6	0.612
Side effect				
BL	0.0	− 0.3	0.3	1
H2	0.5	0.2	0.9	0.004
H24	0.3	− 0.1	0.6	0.208
D6	− 0.2	− 0.6	0.1	0.219
Heart rate (10 × min^−1^)				
BL	0.3	0.07	0.45	0.013
H2	0.1	− 0.2	0.42	0.515
H24	0.4	0.07	0.67	0.023
D6	0.2	0.01	0.43	0.049
Respiratory rate (min^−1^)	− 0.04	− 0.07	0.00	0.037
Core temperature (°C)	0.2	0.0	0.3	0.047
Room temperature (°C)	0.2	0.1	0.4	< 0.001

*CI* 95% confidence interval. Side effect denotes the difference of *T*_S_ between the right and left side of the chest wall

**Table 4:** *T*_S_ in patients with documented angina pectoris.

	${\text{Estimate}}$	CI low	CI high	$p$
CCS 1–3				
H2	− 1.8	− 2.3	− 1.2	< 0.001
H24	− 0.2	− 0.7	0.4	0.587
D6	0,0	− 0.4	0.44	0.946
CCS 4 *vs*. CCS 1–3				
H2	− 1.3	− 2.1	− 0.54	0.002
H24	− 1.6	− 2.44	− 0.68	< 0.001
D6	− 1.8	− 2.53	− 0.98	< 0.001
Heart rate	0.3	0.14	0.39	< 0.001
Systolic blood pressure (10 × mmHg^−1^)	0.1	0.03	0.2	0.01

For patients with CCS1-3, *T*_S_ was compared to baseline. For patients with CCS4, *T*_s_ deviation from patients with CCS1–3 is shown (CCS 4 vs. CCS 1–3)

### Relation Between Chest Wall Temperature and Cutaneous Blood Volume Pulse

**Figure 3:**
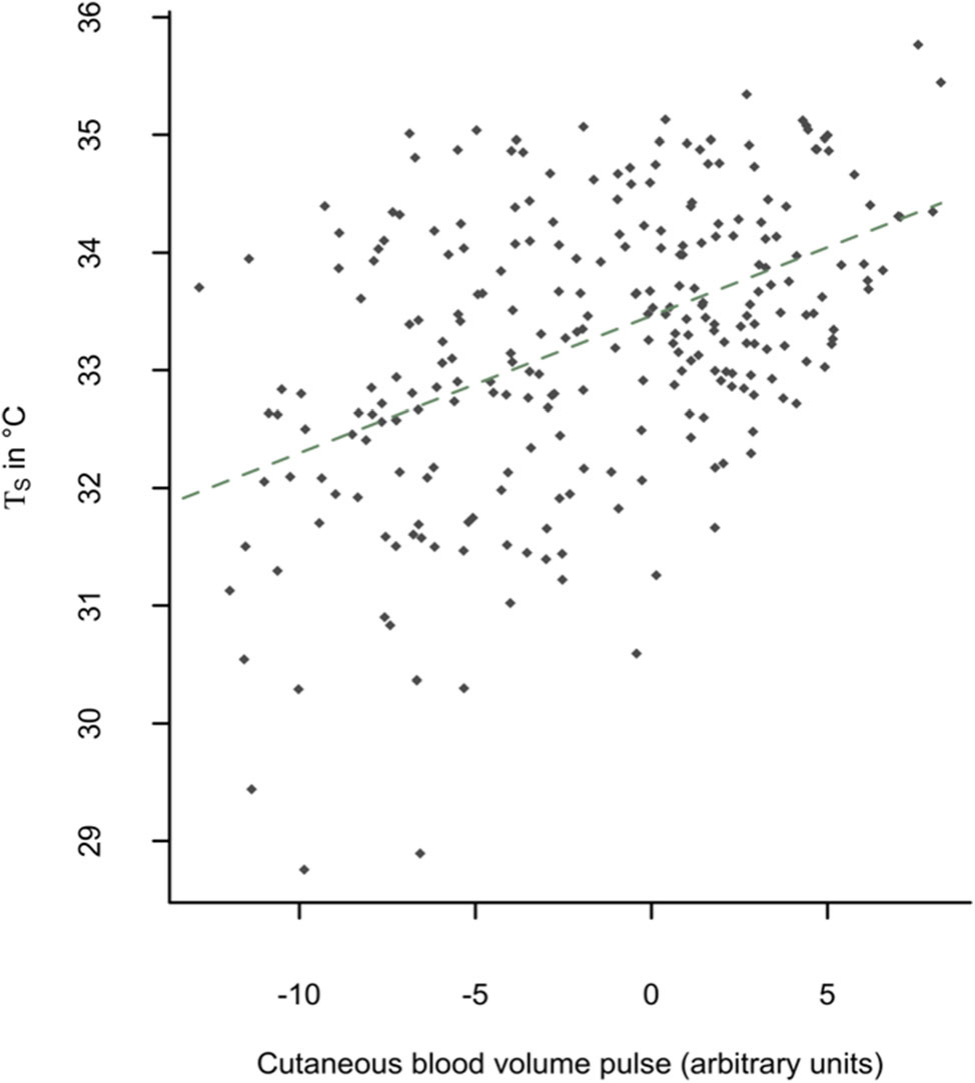
Association between cutaneous blood volume pulse (CVP) and chest wall surface temperature (*T*_S_). The dotted line shows the linear fit of *T*_S_ to CBVP.

*T*_S_ significantly increased with higher CBVP estimated by non-contact photoplethysmography, albeit the association was moderate (Fig. 3). 25.8% of the variability of *T*_S_ was explained by changes of CBVP. Taking into account vital signs and measurement time, the independent effect of CBVP consistently remained. It was not dependent on measurement time (*p* = 0.98 for the interaction).

## Discussion

The present study measured the chest wall surface temperature (*T*_S_) using high resolution thermographic imaging in 42 heart surgery patients. Although expectedly *T*_S_ was correlated to core temperature and room temperature, multivariable analysis confirmed that both temperatures only explained a minor part of *T*_S_ variance and that an independent residual effect of surgery on its own was present. Two hours after surgery, *T*_S_ significantly decreased compared to the preoperative measurement. The expected drop in *T*_S_ on the left compared to the right side, where the main blood supply to the chest wall was removed, was also statistically significant at this point. At the next measurement, 24 h after surgery, the difference between *T*_S_ and core temperature as well as between the chest wall sides had disappeared. While the surgical trauma and the operating room temperature had probably an effect on the core temperature, the further drop in *T*_S_ may be attributed on the one hand to the postoperative peripheral vasoconstriction^21^ and on the other hand to the surgical interruption of the local skin and soft tissue perfusion, which is emphasized on the less perfused left side. Until the next measurement, 24 h after surgery, compensatory mechanisms had gradually reversed these changes, including the difference between sides. Here might come into role the redistribution of circulation with opening of choke vessels, which connect different vascular zones.^3,5^ The fact that *T*_S_ was independent of the heart rate only at H2 sustains the idea, that the local perfusion impairment due to surgery was responsible for the *T*_S_ drop all together and on the left side. After reestablishement of local perfusion, the heart rate could influence again *T*_S_.

The physiological parameters had a minimal impact on *T*_S_. While the respiratory rate was slightly and inversely associated with *T*_S_, blood pressure had an impact on *T*_S_ only in patients with preoperative angina pectoris. The delayed recovery in Class 4 CCS patients may be attributed to a pronounced general vascular injury and successively reduced flow-mediated dilation in these patients, which could delay the opening of choke vessels and the reorganization of circulation.^14^ The absent effects of preoperative ejection fraction and NYHA stage on *T*_S_ are in accordance with the compensated resting hemodynamics of all patients. Although an improvement in ejection fraction is expected within 30 days after CABG^8^ and therefore an improvement in peripheral tissue perfusion, the measurement interval in our study was possibly too short for these changes to take place. Out of the surgical factors, while the use of stapler, gelaspone or intracutaneous sutures were not expected to have a direct impact on tissue perfusion and *T*_S_, the skeletonization of the IMA also didn’t systematically show an impact on *T*_S_. While on the long-term this technique has shown to improve the chest wall perfusion,^6^ the surgical preparation with vessel spasm probably impede the rapid reestablishement of tissue perfusion immediately postoperative. Diabetes mellitus, hypertension and lipid disorders had no influence on *T*_S_, showing that these factors do not affect the rapid circulation changes described above.

The systematic link between *T*_S_ and CBVP proves that the perfusion of superficial skin vessels partly determines *T*_S_. Owing to the limited propagation of light into the skin, CBVP is mainly determined by blood volume changes in the outer skin layers. However, there is only a weak relation between both measures (explanatory power 25.8%). These data support the notion that high resolution thermal imaging depends to some extend on peripheral perfusion, as expected, but also on thermal effects of deeper regions of the chest wall, that are more affected by IMA severance and are critical for wound healing.

The results of this study show, that perfusion alterations after cardiac surgery and even local effects of IMA harvesting can be early detected by means of high resolution IRT. The limited explanatory value of core or room temperature for *T*_S_ emphasizes the specific physiologic significance of IRT monitoring in the context of peripheral perfusion. If the method allows to discriminate between expected and pathological changes in chest wall blood flow, i.e., if it could serve as an early indicator of complications associated to malperfusion, remains to be seen in further studies.

IRT has established itself in the past decades as a non-invasive diagnostic technique. The applications vary from veterinary to human medicine and head to toe.^24^ Around the chestwall, IRT can be used as an adjunctive to mammography to detect breast cancer. The higher tumoral metabolism rate enables the detection within breast thermal maps.^22^ Zhang *et al*.^27^ showed that the temperature difference between the two anterior chest wall sides can reliably predict the succces of the regional anesthetical block. The blockade of small unmyelinated fibers in this case causes a vasodialation and interferes with the physiological thermoregulation, compared to the control side. This sustains the findings of our study, that local changes in blood perfusion have a thermographic effect.

IRT is furthermore used in the mapping of skin perforators in flap surgery. According to the angiosome principle, a perforator vessel supplies blood flow to a vascular territory and has anastomoses to other neighbouring vascular territories.^25^ Currently, dynamic IRT is being used preoperatively in identifying the appropriate perforator vessel to be transplanted as well as in flap monitoring after free flap transplantation, where changes in arterial or venous perfusion influence the skin temperature.^17,23,28^ The sensitivities lie between 89.6 and 99.6% and specificities between 96 and 99.9%.^20^

After supplying blood to the chest wall, IMA continues to the abdomen wall as the superior epigastric artery, where it anastomosises itself to the inferior epigastric artery to supply the perfusion to the abdominal wall. In breast reconstruction using abdominal flaps, the inferior epigastric artery is elevated together with the fasciocutaneous tissues and the vessel anastomosis is usually performed to the IMA and vein. Nergård *et al*.^18^ investigated the abdominal wall perfusion using dynamic IRT in 17 female patients after receiving a breast reconstruction as described before. They recorded a loss of hot spots corresponding to the perforator vessels not only in the undermined median regions, but also in the submammary region on the side where the internal mammary vessels were severed at the level of the third or fourth rib. This sustains the results of the present study, that the harvest of the IMA results in a decline in skin perfusion immediately postoperative. According to Dhar *et al*.,^5^ after a 3 h period of vasoconstriction, the chocke vessels connecting different angiosomes start progressively dilating to reach a normal diameter within 24 h and a maximal dilation at 48–72 h. This was confirmed in the study of Nergård *et al*.,^18^ where the hot spots reappeared within the first postoperative days, as well as in our study, where the chest wall temperature equalized within 24 h of surgery.

The present study was performed in a rather limited amount of patients and did not investigate the impact of harvesting BIMA on the chest wall pefusion. The investigation resumed to a quantitative measurement of the temperature, without identifying the individual perforators. The planning of future studies could include a dynamic investigation with the identification of hot spots. This could show more accurately the impact of thoracotomy and the harvesting of the IMA, as direct perforators could be either closed or show less flow.

In the present study, we showed that the high resolution thermography is suitable to detect early perfusion changes of the chest wall and even local effects of IMA harvesting after CABG. We also showed, that the harvest of LIMA has an additive impact on the hemithoracic blood supply in the first day after surgery. Further studies should aim at establishing a quantitative threshold, in order to predict the wound healing disorders according to the measurement of local blood supply.

## Abbreviations

BIMA: Bilateral internal mammary artery
BL: Before surgery
CABG: Coronary artery bypass graft
CCS: Canadian Cardiovascular Society grading of angina pectoris
CHD: Coronary heart disease
CVP: Cutaneous blood volume pulse
D6: 6 Days after surgery
DSWI: Deep sternal wound infection
H2: Two hours after surgery
H24: 24 Hours after surgery
IMA: Internal mammary artery
LIMA: Left internal mammary artery
LR: Likelihood ratio
LVEF: Ejection fraction
NUC: Non-uniformity correction
NYHA: New York heart Association functional class
ped: Pedicled mammaria graft
PPGI: Photoplethysmographic imaging
ROI: Regions of interest
scel: Skeletonized mammaria graft
Ts: Chest wall surface temperature
T_S,L_: Left chest wall surface temperature
T_S,R_: Right chest wall surface temperature
